# Review of cardiac safety in onasemnogene abeparvovec gene replacement therapy: translation from preclinical to clinical findings

**DOI:** 10.1038/s41434-023-00401-5

**Published:** 2023-04-25

**Authors:** Deepa H. Chand, Rui Sun, Karim A. Diab, Damien Kenny, Francis Fonyuy Tukov

**Affiliations:** 1 Novartis Pharmaceuticals Corporation, East Hanover, NJ USA; 2 Department of Pediatrics, University of Illinois College of Medicine and Children’s Hospital of Illinois, Peoria, IL USA; 3 Division of Cardiology, Department of Pediatrics, Inova Children’s Hospital, Fairfax, VA USA; 4 Department of Paediatric Cardiology, CHI at Crumlin, Dublin, Ireland

**Keywords:** Biomarkers, Biomarkers

## Abstract

Human gene replacement therapies such as onasemnogene abeparvovec (OA) use recombinant adeno-associated virus (rAAV) vectors to treat monogenic disorders. The heart and liver are known target organs of toxicity in animals; with cardiac and hepatic monitoring recommended in humans after OA dosing. This manuscript provides a comprehensive description of cardiac data from preclinical studies and clinical sources including clinical trials, managed access programs and the post-marketing setting following intravenous OA administration through 23 May 2022. Single dose mouse GLP-Toxicology studies revealed dose-dependent cardiac findings including thrombi, myocardial inflammation and degeneration/regeneration, which were associated with early mortality (4-7 weeks) in the high dose groups. No such findings were documented in non-human primates (NHP) after 6 weeks or 6 months post-dose. No electrocardiogram or echocardiogram abnormalities were noted in NHP or humans. After OA dosing, some patients developed isolated elevations in troponin without associated signs/symptoms; the reported cardiac adverse events in patients were considered of secondary etiology (e.g. respiratory dysfunction or sepsis leading to cardiac events). Clinical data indicate cardiac toxicity observed in mice does not translate to humans. Cardiac abnormalities have been associated with SMA. Healthcare professionals should use medical judgment when evaluating the etiology and assessment of cardiac events post OA dosing so as to consider all possibilities and manage the patient accordingly.

## Introduction

Human gene therapies using recombinant adeno-associated virus (rAAV) vectors are being studied for a wide variety of monogenic disorders including spinal muscular atrophy (SMA) [1, 2]. Clinically, children affected with SMA demonstrate loss of motor neurons leading to progressive loss of muscle strength and function; difficulties in swallowing and breathing; and, possibly death [3]. Although the primary pathology of SMA is neurodegeneration at the spinal motor neuron, some clinical reports indicate involvement of other organs including heart and liver [4, 5]. Winjaarde et al. presented a comprehensive review of animal and human data with respect to cardiac findings associated with SMA from 14 publications from SMA mouse studies and 72 studies of 264 patients with SMA. Cardiac pathology was noted in 77 patients with Type 1 SMA, including conduction abnormalities in 33/77 (43%); and structural abnormalities in 42/77 (55%) of patients. Of the 42 patients with structural abnormalities, 21 (50%) had multiple defects and 21 had a single defect, with the most common being atrial septal defect (ASD), ventricular septal defect (VSD), and hypoplastic left heart syndrome [5]. Cardiac findings in SMA mouse models included low left ventricular mass, myocardial fibrosis, vascular remodeling, and a numerical decrease in coronary capillaries [5].

Onasemnogene abeparvovec (OA) is a gene replacement therapy product that encodes for the human survival motor neuron (hSMN) protein, the inadequate production of which appears to be the root cause of SMA. Onasemnogene abeparvovec is comprised of a non replicating, non integrating recombinant self complementary adeno associated virus serotype 9 (AAV9) capsid shell containing the cDNA of the human SMN gene [6]. Onasemnogene abeparvovec contains no DNA from the wild type AAV9, rendering it incapable of replicating itself. Hence, the AAV9 is used to deliver the respective transgene required to treat the underlying disease state.

Preclinical murine data with OA identified the heart as a target organ of toxicity with histopathologic evidence of inflammation and intracardiac thrombi [6]. Therefore, cardiac adverse events have been selected as an adverse events of special interest in all clinical trials and post-marketing safety surveillance.

Clinical trials included cardiac troponin I, electrocardiogram (ECG), and echocardiogram assessments. Current recommendations outline monitoring cardiac troponin-I before OA infusion and on a regular basis for at least 3 months afterwards [7]. There are no recommendations for routine ECG or echocardiogram monitoring.

This manuscript provides a comprehensive description of cardiac safety data from preclinical studies, clinical trials and post-marketing reports from all children treated with OA through 23 May 2022.

## Materials and methods

### Preclinical studies

Nonclinical safety studies using single-dose intravenous (IV) administration of comparable lots of clinical grade OA to neonatal mice and juvenile non-human primates (NHP; cynomolgus monkeys) were undertaken to determine safety risks possibly relevant for patients treated with OA. Principles of the International Council of Harmonisation (ICH) M3 (R2) Guideline on Nonclinical Safety Studies for the Conduct of Human Clinical Trials for Pharmaceuticals, ICH S6 (R1) Preclinical Safety Evaluation of Biotechnology-Derived Pharmaceuticals and US Food and Drug Administration Guidance for Industry were applied. These studies were performed in accordance with Good Laboratory Practice guidelines at contract research organizations (CROs) using a Good Manufacturing Practices‒compliant test article representative of the pivotal clinical trial and commercial drug product. For the reported mouse studies, animal welfare guidelines of the CROs contracted to perform the studies were used as defined the study protocols/plans. All study procedures in the Protocols complied with the Animal Welfare Act, the Guide for the Care and Use of Laboratory Animals, and the Office of Laboratory Animal Welfare and were approved by the local institutional Animal Care and Use Committee (IACUC).

Juvenile NHPs (cynomolgus monkeys) and neonatal mice (FVB/NJ mice) were used because of their established relevance as experimental toxicology species and their permissiveness to AAV9 transduction. The current clinical dose (1.1 × 10^14^ vg/kg) for OA was employed in the NHP study. Doses tested in the mouse studies ranged from 7.9 × 10^13^ to 3.9 × 10^14^ vg/kg, and clearly characterized the full toxicity dose response from below the clinical equivalent dose based on weight (1.1 × 10^14^ vg/kg) up to and beyond the maximum tolerated dose (MTD, 1.5 × 10^14^ vg/kg).

### Cardiac toxicity assessment in mice

Cardiac toxicity was evaluated in two 12-week GLP-compliant mouse studies designed to assess potential toxicity of OA when administered once via temporal vein injection in neonatal FVB/NJ mice. FVB/NJ mice is an inbred strain of mouse model that is most commonly used for transgenic applications, and has a reasonably normal lifespan under lab conditions, with 60% living beyond two years. There does not appear to be any published evidence that this mouse strain has cardiac involvement as part of its natural history.

### Study 1 (a single dose temporal vein injection study in the neonatal FVB/NJ mouse followed by a 12-week observation period)

#### Animals

Male and female FVB/NJ mice (F_0_) were received at age 9 weeks. The mated dams delivered naturally at the Testing Facility.

#### Study design

Four groups of 45 one-day old (post-natal day (PND)1) FVB/NJ mice/sex were dosed once via IV, slow bolus injection in the temporal vein with OA at 7.9 × 10^13^ vg/kg, 2.37 × 10^14^ vg/kg or 3.91 × 10^14^ vg/kg or control 0.9% saline. Mice in all dose groups were designated for general toxicology assessment, clinical pathology and histological analysis at weeks 3, 6 and 12. In-life assessments included daily or weekly general viability evaluations; evaluations between one and 3 h post-dose; and body weights on the day of dosing, twice weekly through PND 28 and weekly thereafter. Blood samples for clinical pathology were collected terminally at weeks 3, 6 and 12. At weeks 3, 6, and 12, the first five mice/sex/group were euthanized, subjected to gross necropsy and organ weighing. Heart tissue was collected, embedded in paraffin, sectioned, mounted on glass slides, and stained with hematoxylin and eosin. Histopathology evaluation was completed for a select panel of these tissues including but not limited to gross lesions, brain, heart, kidney, liver, lung, skeletal muscle, spinal cord, etc., by a board-certified veterinary pathologist.

### Study 2 (a single dose of AVXS-101 via temporal vein injection in neonatal FVB/NCrl mice followed by a 12-week observation period)

#### Animals

One hundred F_0_ time-mated female FVB/NCrl mice were received from the supplier. The females arrived on or prior to Gestation Day (GD) 12 and delivered litters (F_1_ offspring) at the test facility, Covance (now Labcorp) Laboratories Inc., Greenfield, IN, USA. F_0_ animals were allowed to acclimate for at least 3 days.

#### Study design

Four groups of male and female neonatal FVB/NCrl mice (47-49/sex/group) were administered vehicle control article (0.9% saline) or OA at 1.5 × 10^14^, 2.4 × 10^14^, or 3.0 × 10^14^ vg/kg IV via single temporal vein injection once on PND 0. F_1_ offspring (192 males and 192 females) weighed 1.1–1.7 g on the day (PND 0) of dosing. The vehicle control article was OAV101 Formulation buffer (20 mM Tris, 1 mM MgCl2, 200 mM NaCl, 0.005% Poloxamer 188, pH 8.0 ± 0.1 at 20 °C), also known as TOX Control (TFF3 Buffer). Mice in all dose groups were designated for general toxicology assessment, clinical pathology and histological analysis and were sacrificed in week 3, 6 or 12 post-dosing. In-life assessments included twice daily general viability evaluations and weekly detailed observations performed in conjunction with body weight and food consumption measurements.

#### Safety assessment

Blood samples for clinical pathology were collected at weeks 3, 6 and 12, when the first five mice/sex/group were euthanized, subjected to gross necropsy and organ weighing. A comprehensive panel of tissues including the heart was collected, embedded in paraffin, sectioned, mounted on glass slides, and stained with hematoxylin and eosin. Histopathology evaluation was completed for a select panel of these tissues including but not limited to gross lesions, brain, heart, kidney, liver, lung, skeletal muscle, spinal cord, by a board-certified veterinary pathologist, and a formal contemporaneous pathology peer reviews were conducted by the Sponsor selected peer review pathologists. Digital scans (virtual slides) of heart lesions and selected microscopic slides were collected at necropsy and sent to the Sponsor for illustrative purposes.

### Cardiac toxicity assessment in Non-Human Primates (NHP)

Cardiac toxicity was evaluated in a 6-month NHP study designed to evaluate the toxicity of OA when administered as a single dose IV infusion to juvenile cynomolgus monkeys with or without immunosuppressant (prednisolone) treatment.

#### Animals

Cynomolgus monkeys (*Macaca fascicularis*) of Asian origin were received up to 10 weeks prior to dosing. Animals were 14 to 17 months old and weighed 1.5 to 2.0 kg (males) and 1.3 to 1.9 kg (females) at the initiation of dosing.

#### Study design

Three groups of cynomolgus monkeys (*n* = 6 animals/sex/group) were administered vehicle control article or 1.1 × 10^14^ vg/kg OA by IV infusion via saphenous vein over approximately 20 minutes using a calibrated external pumping device in the presence or absence of prednisolone (1.0 mg/kg/day, per os). Animals were euthanized at 6 weeks post dose (3 animals/sex/group) or at 6 months post dose (2 animals/sex/group). Clinical observations, assessments of body weight and qualitative food consumption; electrocardiogram (ECG) measurements were performed. Electrocardiograms were recorded on conscious, restrained animals once during the predose phase and once during Weeks 1, 6, 13, and 26 of the dosing phase. Blood was collected for Cardiac troponin I (cTnI) analysis from fasted animals via the femoral vein once during the predose phase and once on Days 8, 15, 22, 36, 85, 127, and 176 of the dosing phase. Tissues from each animal including macroscopic lesions and the heart were collected for macroscopic and microscopic pathology evaluations. Tissues for microscopic evaluation were collected, embedded in paraffin, sectioned, and slides prepared and stained with hematoxylin and eosin (H&E), which were examined microscopically by the board certified Anatomic Pathologist. A pathology peer review evaluation was performed following completion of the primary microscopic evaluation.

### Clinical trials

Five interventional clinical trials (Table 1), in which children with SMA were dosed with IV OA have been completed. All studies were performed in accordance with ethical principles in the Declaration of Helsinki and are consistent with ICH/Good Clinical Practice and applicable regulatory requirements. All study protocols were approved by the institutional review boards or ethics committees, with appropriate informed consent obtained. Three patients received a low dose of OA (6.7 × 10^13^ vg/kg), after which the dosing was increase to 1.1 × 10^14^ vg/kg IV, which is considered the therapeutic dose. Patients who received this therapeutic dose in clinical trials are included in this analysis.

**Table 1:** Completed OA IV clinical trials.

Study number phase country(ies)	Study design/route of administration/duration of treatment	Number of subjects	Diagnosis of patients	SMA type (*SMN2* Copy No.)	NCT number
START Phase 1 United States	Open-label, single-center IV Single dose	15	Patients with SMA Type 1 with 2 copies of SMN2 < 6 months of age at the time of gene replacement therapy.	1 (2)	NCT02122952
STR1VE Phase 3 United States	Open-label, single-arm, multicenter IV Single dose	22	Patients with SMA Type 1 with 1 or 2 copies of *SMN2* < 6 months of age at the time of gene replacement therapy	1 (1,2)	NCT03306277
SPR1NT Phase 3 Global (multi-countries)	Open-label, single-arm, multicenter IV Single dose	29	Pre-symptomatic patients with genetically confirmed SMA Type 1 or 2 with 2 or 3 copies of *SMN2*, ≤ 6 weeks of age at the time of gene replacement therapy	1, 2 (2, 3, 4)	NCT03505099
STR1VE-EU Phase 3 Europe	Open-label, single-arm, multicenter IV Single dose	33	Patients with SMA Type 1 with 1 or 2 copies of *SMN2* < 6 months of age at the time of gene replacement therapy	1 (1,2)	NCT03461289
STR1VE-AP Phase 3 Asia	Open-label, single-arm, multicenter IV Single dose	2	Patients with SMA Type 1 with 1 or 2 copies of *SMN2* < 6 months of age at the time of gene replacement therapy	1 (1, 2)	NCT03837184

Cardiac data evaluated for each study included reported adverse events (AEs), vital signs, cardiac troponin (using any assay), ECG, and transthoracic echocardiography results. AEs that were classified as cardiac AEs were determined by Standardised MedDRA Queries (SMQs, all broad search): Cardiomyopathy, Ischemic heart disease, Cardiac arrhythmias, Embolic and thrombotic events, Myocardial infarction, and Cardiac failure, and Hypertension. Cardiac systolic function was assessed using results for left ventricular ejection fraction (LVEF) and left ventricular fractional shortening (LVFS), for which normative values were considered to be between 56% and 78%; for LVEF and between 28% and 46% for LVFS [8].

### Data from long term follow up studies, registry (including US Managed Access Program [US MAP]), and post-marketing reports

Two long-term follow up studies (including patients dosed in the IV parent studies in Table 1) and a registry are ongoing and all reported cardiac AEs are captured in the safety database. Patients from the START study (*n* = 13) and as well as the other 4 studies (*n* = 59) were rolled over to one of two long-term follow up studies.

The Novartis global safety database was searched cumulatively through 23 May 2022 for all individual case safety reports (ICSRs) received from the long-term follow up studies, the registry and all sources of post-marketing reports. Cardiac AEs were identified using the same SMQs as used for the clinical trials. A sub-analysis was conducted to evaluate if cardiac events coincided with hepatic events, since the liver is also a target organ of toxicity for OA. Hepatic events were identified by the SMQ Hepatic disorders.

## Results

### Preclinical studies

#### Six-month NHP IV study

Administration of 1.1 × 10^14^ vg/kg OA with or without prednisolone to juvenile cynomolgus monkeys via a single IV infusion was generally well tolerated for up to 6 months post IV infusion and was not associated with mortality, clinical observations, or any pathology (gross or microscopic) findings in the heart at 6 week or 6 month postdose.

No OA-related increases were observed in plasma cTnI. Any other differences in plasma cTnI, compared to baseline and/or control data, were not attributed to test article because they were sporadic, consistent with biologic variation, negligible in magnitude, and/or lacked dose relationship. Additionally, no changes in PR interval, QRS duration, QT interval, corrected QT (QTc) interval, heart rate, and abnormal waveforms or arrhythmias were observed during Week 1, 6, 13, or 26 in any of the animals dosed with or without prednisolone.

#### 12-week GLP mouse studies

##### Study 1 (a single dose temporal vein injection study in the neonatal FVB/NJ mouse followed by a 12-week observation period)

Administration of a single IV dose of OA to PND1 FVB/NJ mice was clinically tolerated at doses of 7.9 × 10^13^ and 2.37 × 10^14^ vg/kg. Moribundity and unscheduled mortality were recorded in mice administered 3.91 × 10^14^ vg/kg, primarily during the weeks 4-7 of the study. Of the 29 mice presented for examination at unscheduled intervals, morbidity/death of 13 mice was attributed to OA-related atrial thrombi in the heart (Table 2). Thrombi were not observed macroscopically in the heart, but noted microscopically and were characterized by variable collections of fibrin, cellular debris, and inflammatory cells in the atria/auricles that were often adjacent to or attached to plump, reactive endothelium. No evidence of thrombus or embolization was noted in any other organ. The cause of morbidity/death for the remaining 16 OA-treated mice examined that died prior to scheduled sacrifice was undetermined. In addition, while no gross/macroscopic lesions were noted in the heart of any of the preterminal mice whose moribundity/mortality were attributed to the test article, these mice were noted with other microscopic heart findings including minimal or mild microscopic degeneration/regeneration in the heart. These findings, together with thrombi in the heart, may have contributed to the deteriorating clinical condition. OA-related mortality and adverse clinical observations and body weight changes did not occur at doses ≤2.37 × 10^14^ vg/kg.

**Table 2:** Summary of microscopic findings in mice-early deaths.

	Males				Females			
Group	1	2	3	4	1	2	3	4
Virus Concentration (Vg/kg)	0	7.9 × 10^13^	2.37 × 10^14^	3.91 × 10^14^	0	7.9 × 10^13^	2.37 × 10^14^	3.91 × 10^14^
No. Animals	1	0	0	16	0	0	0	13
Heart (No. Examined)	(1)^a^	(0)	(0)	(16)	(0)	(0)	(0)	(13)
Thrombus	0	0	0	8	0	0	0	11
Minimal	–	–	–	1	–	–	–	3
Mild	–	–	–	2	–	–	–	4
Moderate	–	–	–	5	–	–	–	4
Degeneration/regeneration	0	0	0	12	0	0	0	13
Minimal	–	–	–	4	–	–	–	2
Mild	–	–	–	8	–	–	–	11

^a^Numbers in parentheses represent the number of animals with the finding.

– = Not applicable

OA-related microscopic findings in the heart at scheduled sacrifice intervals comprised of degeneration/regeneration observed at 3-, 6- and/or 12-weeks postdose across all treatment groups, and thrombi at week 6 in mice dosed at 3.91 × 10^14^ vg/kg. Due to the unscheduled mortality up to weeks 6/7 and the scheduled euthanasia at week 3 and weeks 6/7, no mice administered 3.91 × 10^14^ vg/kg were available for the week 12 time point. The thrombi in the heart appeared morphologically similar to those described in the mortality section above. The degeneration/regeneration was characterized by shrunken, wavy cardiomyocytes and/or clear spaces between cardiomyocytes (degeneration), associated slight mononuclear cell hypercellularity, crowded residual myocyte nuclei, or occasional cardiomyocyte nuclei that were enlarged 2–4 times those of adjacent cells (regeneration). In general, the change was most prominent in the myocardium near the endocardial (luminal) surfaces of the left and right ventricular free walls, interventricular septum, and bases of papillary muscles, although the atrial myocardium was also sometimes involved. At week 12 the heart degeneration/regeneration was minimal, in contrast to mild heart degeneration/regeneration seen in some mice at weeks 3 and 6/7 (less severe overall at week 12 than at other time-points), which was consistent with repair/healing over time (Table 2).

At 3-weeks postdose, degeneration//regeneration in the heart was seen at all doses with severity being mild at doses ≥2.37 × 10^14^ vg/kg, and minimal at 7.9 × 10^13^ vg/kg, which supported a dose-response. While still present at 6 week postdose at minimal or mild severity, only one mouse administered 7.9 × 10^13^ vg/kg was noted with degeneration//regeneration in the heart. The finding was noted more at doses ≥2.37 × 10^14^ vg/kg with average severities in these groups being slightly less than the average severities of the same groups at week 3, possibly showing a trend toward lesion resolution over time. Thrombi (moderate severity) in the heart were present only in mice administered 3.91 × 10^14^ vg/kg at this interval. At 12 weeks postdose, degeneration//regeneration in the heart was noted in mice administered 7.9 × 10^13^ or 2.37 × 10^14^ vg/kg at a severity of minimal, in contrast to mild changes seen at weeks 3 and 6/7. This indicated a trend toward lesion resolution over time.

Minimal to mild clinical pathology changes consistent with possible inflammation (increased lymphocytes, monocytes, eosinophils and/or neutrophils, decreased reticulocytes) were observed at all dose levels as early as Week 3. Increased creatine kinase activity was noted as early as Week 3 in mice given 3.91 × 10^14^ vg/kg and then at later timepoints in mice dosed at ≥7.9 × 10^13^ vg/kg. These changes were possibly associated with the microscopic findings of degeneration/regeneration in the heart.

##### Study 2 (a single dose of AVXS-101 (Lot Number 600443) via temporal vein injection in neonatal FVB/NCrl mice followed by a 12-week observation period)

Administration of OA in mice resulted in mortality between weeks 4 and 12 and when the cause of death could be ascribed, it was associated with atrial thrombosis at ≥2.4 × 10^14^ vg/kg. OA-related microscopic findings were dose-dependent, noted in the atria and ventricular myocardium and mostly in mice administered ≥2.4 × 10^14^ vg/kg. OA-related ventricular myocardial findings included minimal to slight myocardial degeneration/necrosis, and/or minimal to slight mononuclear cell inflammation, whereas, the atrial findings consisted of minimal to moderate thrombus formation, slight to marked dilation, minimal to slight fibroplasia. Atrial thrombosis and associated atrial wall changes were dose-related and present at higher incidences and/or severities in unscheduled death animals administered ≥2.4 × 10^14^ vg/kg, which suggested that atrial lesions may have been associated with morbidity (in moribund sacrifice animals) and mortality. The microscopic findings in the heart were sometimes associated with macroscopic findings of large size, abnormal shape, and/or large atrium, and increased heart weights in some animals administered 3.0 × 10^14^ vg/kg. There was no evidence of thrombi in the cardiac chambers upon gross pathology assessments. The only OA-related hematology finding was minimally higher neutrophil count on PND 42 and 84 in males administered ≥2.4 × 10^14^ vg/kg, suggestive of an inflammatory response (Tables 3–7).

**Table 3:** Summary of microscopic findings in mice—terminal euthanasia (weeks 3, 6/7 and 12).

	Males				Females			
Group	1	2	3	4	1	2	3	4
Test article virus concentration (vg/kg)	0	7.9 × 10^13^	2.37 × 10^14^	3.91 × 10^14^	0	7.9 × 10^13^	2.37 × 10^14^	3.91 × 10^14^
No. Animals Examined	**5**	**5**	**5**	**5**	**5**	**5**	**5**	**5**
Heart (No. Examined)	(5)^a^	(5)	(5)	(5)	(5)	(5)	(5)	(5)
Week 3								
No. Animals Examined	(5)^a^	(5)	(5)	(5)	(5)	(5)	(5)	(5)
Degeneration/regeneration	0	3	5	5	1	5	5	5
Minimal	–	3	0	0	1	5	0	0
Mild	–	0	5	5	0	0	5	5
Week 6/7								
Heart (No. Examined)	(5)^a^	(5)	(5)	(4)	(5)	(5)	(5)	(5)
Degeneration/regeneration	0	1	5	4	0	0	5	5
Minimal	–	1	4	2	–	–	1	0
Mild	–	0	1	2	–	–	4	5
Thrombus	0	0	0	1	0	0	0	1
Moderate	–	–	–	1	–	–	–	1
Week 12								
Heart (No. Examined)	(5)^a^	(5)	(5)	(0)	(5)	(5)	(5)	(0)
Degeneration/regeneration	0	2	5	–	0	2	5	–
Minimal	–	2	5	–	–	2	5	–

As this is a gene therapy that is administered using a viral vector, the amount of virus contained in the product is very important. The bolded row represents that viral concentration.

^a^Numbers in parentheses represent the number of animals with the finding.

- = Not applicable

**Table 4:** Incidence and severity of OA-related microscopic findings in mice—unscheduled sacrifices.

Sex	OA							
	Males^a^				Females^b^			
Test article virus concentration (vg/kg)	Vehicle control	1.5 × 10^14^ vg/kg	2.4 × 10^14^ vg/kg	3.0 × 10^14^ vg/kg	Vehicle control	1.5 × 10^14^ vg/kg	2.4 × 10^14^ vg/kg	3.0 × 10^14^ vg/kg
Heart								
Number examined	1	0	1	5	2	1	2	5
Inflammation, mononuclear, ventricle								
Minimal	0	NE	1	5	0	0	1	2
Edema, ventricle								
Minimal	0	NE	1	3	0	1	0	0
Fibrosis, myocardium, ventricle								
Minimal	0	NE	1	5	0	0	2	4
Thrombus, atrium								
Minimal	0	NE	0	0	0	0	1	2
Slight	0	NE	0	1	0	0	1	1
Moderate	0	NE	0	2	0	0	0	1
Dilation, atrium								
Slight	0	NE	0	2	0	0	1	2
Moderate	0	NE	0	1	0	0	0	0
Marked	0	NE	0	1	0	0	0	0
Fibroplasia, atrium								
Minimal	0	NE	0	1	0	0	1	2
Slight	0	NE	0	3	0	0	1	2
Degeneration/necrosis, myocardium, atrium								
Minimal	0	NE	0	3	0	0	0	2
Slight	0	NE	0	1	0	0	2	3
Inflammation, mononuclear, atrium								
Minimal	0	NE	0	4	0	0	1	4

*NE n*ot examined.

^a^One male (Animal M0218-2) administered 2.4 × 10^14^ vg/kg was not examined microscopically due to extensive autolysis.

^b^One control female (Animal M0020-6) was not examined microscopically due to extensive autolysis.

**Table 5:** Incidence and severity of OA-related microscopic heart findings in mice—week 3 (PND 21) terminal sacrifice.

Sex	OA							
	Males				Females			
Test article virus concentration (vg/kg)	Vehicle control	1.5 × 10^14^ vg/kg	2.4 × 10^14^ vg/kg	3.0 × 10^14^ vg/kg	Vehicle control	1.5 × 10^14^ vg/kg	2.4 × 10^14^ vg/kg	3.0 × 10^14^ vg/kg
Heart								
Number examined	5	5	5	5	5	5	5	5
Inflammation, mononuclear, ventricle								
Minimal	0	4	5	3	0	3	5	3
Slight	0	0	0	2	0	0	0	2
Edema, ventricle								
Minimal	0	0	4	2	0	1	3	3
Fibrosis, myocardium, ventricle								
Minimal	0	0	3	3	0	3	5	3
Slight	0	0	0	0	0	0	0	2
Inflammation, mononuclear, atrium								
Minimal	0	0	0	1	0	0	0	2

**Table 6:** Incidence and severity of OA-related microscopic heart findings in mice—week 6 (PND 42) terminal sacrifice.

Sex	OA							
	Males				Females			
Test article virus concentration (vg/kg)	Vehicle control	1.5 × 10^14^ vg/kg	2.4 × 10^14^ vg/kg	3.0 × 10^14^ vg/kg	Vehicle control	1.5 × 10^14^ vg/kg	2.4 × 10^14^ vg/kg	3.0 × 10^14^ vg/kg
Heart								
Number examined	5	5	5	5	5	5	5	5
Inflammation, mononuclear, ventricle								
Minimal	0	3	5	5	0	2	5	3
Edema, ventricle								
Minimal	0	4	1	3	0	3	4	4
Fibrosis, myocardium, ventricle								
Minimal	0	2	2	5	0	1	4	5
Thrombus, atrium								
Slight	0	0	1	0	0	0	0	0
Moderate	0	0	0	2	0	0	0	0
Dilation, atrium								
Slight	0	0	0	1	0	0	0	0
Fibroplasia, atrium								
Minimal	0	0	1	2	0	0	0	0
Degeneration/necrosis, myocardium, atrium								
Minimal	0	0	0	3	0	0	0	0
Inflammation, mononuclear, atrium								
Minimal	0	1	1	3	0	0	2	3
Slight	0	0	0	1	0	0	0	0
Slight	0	0	0	1	0	0	0	1

**Table 7:** Incidence and severity of OA-related microscopic heart findings in mice—week 12 (PND 84) terminal sacrifice.

Sex	OA							
	males				Females			
Test article virus concentration (vg/kg)	Vehicle control	1.5 × 10^14^ vg/kg	2.4 × 10^14^ vg/kg	3.0 × 10^14^ vg/kg	Vehicle control	1.5 × 10^14^ vg/kg	2.4 × 10^14^ vg/kg	3.0 × 10^14^ vg/kg
Number Examined	5	5	5	5	5	5	5	5
Inflammation, mononuclear, ventricle								
Minimal	0	4	3	2	0	3	1	0
Edema, ventricle								
Minimal	0	1	1	0	0	1	0	0
Fibrosis, myocardium, ventricle								
Minimal	0	3	5	5	0	5	5	4
Slight	0	0	0	0	0	0	0	1
Thrombus, atrium								
Slight	0	0	0	1	0	0	0	0
Dilation, atrium								
Moderate	0	0	0	1	0	0	0	0
Fibroplasia, atrium								
Minimal	0	0	0	1	0	0	0	0
Slight	0	0	0	1	0	0	0	0
Degeneration/necrosis, myocardium, atrium								
Minimal	0	0	0	1	0	0	0	0
Slight	0	0	0	1	0	0	0	0
Inflammation, mononuclear, atrium								
Minimal	0	1	4	2	0	1	1	1

##### Clinical trial data

A total of 101 patients were dosed with IV OA during the clinical trials; of whom 99 received the therapeutic dose and are included in this analysis. The remaining 3 patients did not receive a dose lower than the therapeutic dose, which was not developed further.

##### Adverse events

Cardiac adverse events by preferred term are presented in Table 7. Cardiac AEs were reported in 17 patients (17.2%) and consisted mainly of elevated enzymes (cardiac troponin and CK-MB) and changes in heart rate (tachycardia and bradycardia) without clinical significance. All other AEs as presented in Table 8 were considered to be related to the underlying SMA, not a cardiac etiology. No adverse events of cardiac thrombi were reported. No patient died of a cardiac AE.

**Table 8:** Summary of treatment-emergent cardiac adverse events by preferred term.

Preferred Term	START	STR1VE-EU	STR1VE	SPRINT	STR1VE-AP	Total
	(*N* = 15) n (%)	(*N* = 33) n (%)	(*N* = 22) n (%)	(*N* = 29) n (%)	(*N* = 2) n (%)	(*N* = 101) n (%)
Total	3 (20.0)	5 (15.2)	4 (18.2)	5 (16.7)	0	17 (16.7)
Blood creatine phosphokinase MB increased	0	1 (3.0)	2 (9.1)	3 (10.0)	0	6 (5.9)
Tachycardia	2 (13.3)	2 (6.1)	1 (4.5)	0	0	5 (4.9)
Troponin increased	0	0	0	3 (10.0)	0	3 (2.9)
Bradycardia	1 (6.7)	1 (3.0)	0	0	0	2 (1.9)
Dyspnea	1 (6.7)	1 (3.0)	0	0	0	2 (1.9)
Troponin T increased	0	2 (6.1)	0	0	0	2 (1.9)
Blood creatine phosphokinase increased	0	0	0	1 (3.3)	0	1 (1.0)
Blood pressure diastolic decreased	0	0	1 (4.5)	0	0	1 (1.0)
Blood pressure systolic increased	0	0	1 (4.5)	0	0	1 (1.0)
Device occlusion	0	1 (3.0)	0	0	0	1 (1.0)
Loss of consciousness	0	1 (3.0)	0	0	0	1 (1.0)

##### Echocardiogram and electrocardiogram

With respect to echocardiogram data, baseline LVEF was normal or borderline high for all but 2 patients who had borderline low values. Two patients each had a single post-treatment occurrence of LVEF values that were slightly below the normal range (51.0% and 55.4%, respectively) which were not considered clinically meaningful. At baseline, the mean (±SD) LVEF was 65.1% (±5.19), and the median LVEF was 64.9% (*N* = 85). Six months after treatment (*N* = 73), the mean LVEF was 68.8% (±5.90), and the median LVEF was 69.3%. At 12 months (*N* = 56) and 18 months (*N* = 23), mean LVEF was similar to the means at baseline and at 6 months after infusion. Baseline LVFS was normal or borderline high for all patients except for a borderline low value (27.1%) in one patient from SPRINT. No patient had a low post-treatment LVFS value. At baseline, the mean (±SD) LVFS was 36.1% (±4.37) and the median LVFS was 35.4%. Six months after treatment the mean LVFS was 38.5% (±4.83) and the median LVFS was 38.0%. At 12 months and 18 months post dosing, the mean LVFS values were similar to the means at baseline and at 6 months after dosing. Upon review of the echocardiogram results, no patients had evidence of cardiac thrombi.

No patient in any of the clinical trials was reported to have persistent potentially clinically significant ECG abnormalities, nor were any findings associated with abnormal clinical signs or symptoms. No abnormalities in PR interval, QRS complex or QTc were present.

##### Laboratory data

Cardiac troponin I (cTnI) as a marker of potential cardiac toxicity was collected in START, and was not collected initially in STR1VE-EU, STR1VE-AP, or SPRINT; however, these protocols were amended to include measurement of cTnI in patients enrolled following the amendment. Cardiac troponin I was not collected in STR1VE. A total of 22 patients had at least one cardiac troponin value obtained and 11/101 (10.9%) patients had baseline values obtained, however, not all patients had cTnI performed; some had cardiac troponin I and/or T performed at local laboratories as compared to central laboratory as this was allowed per protocol. Therefore, the data presented is a mix of high sensitive and normal cTn values. A value of >0.05 ng/mL was considered elevated; the % changes from baseline could not be calculated due to many missing values. The limited data show that cardiac troponin was elevated in some patients at baseline; while in other patients, some increases were seen after treatment with OA and all but one elevation normalized by the Month 2 visit (Table 9). Due to limitations associated with archival, cardiac troponin values from START are not available.

**Table 9:** Summary of patients with document troponin values in OA clinical trials.

Patient ID	Troponin value (ng/mL)										
	Study visit										
	Screening	Day 7	Month 1	Month 2	Month 6	Month 9	Month 12	Month 15	Month 18	Month 21	Month 24
1	0.098	0.114	0.121	0.026	0.005	0.005	0.005	0.005	0.005		
2	0.067	0.042	0.04	0.025	0.021	0.005	0.006	0.006	0.006		
3	0.02	0.02	0.06	0.06	0.02	ND	ND	0.02	0.02		
4	0.02	0.03	0.02	0.02	ND	ND	ND	0.02	0.02		
5	ND	0.02	ND	ND	ND	ND	ND	ND	ND		
6	ND	ND	ND	ND	ND	ND	ND	ND	0.02		
7	0.02	0.04	0.06	0.04	0.02	ND	ND	0.02	0.02		
8	ND	ND	ND	ND	ND	ND	ND	ND	0.02		
9	ND	ND	ND	ND	ND	ND	ND	ND	0.02		
10	ND	ND	ND	ND	ND	ND	ND	ND	0.02		
11	ND	ND	ND	ND	ND	ND	ND	ND	0.02		
12	0.028	0.03	ND	ND	0.02	ND	0.02	0.02	0.02	0.02	0.02
13	0.03	0.04	0.04	ND	0.02	0.02	ND	0.02	0.02	0.02	ND
14	ND	ND	ND	ND	0.02	0.03	ND	ND	ND	ND	0.02
15	ND	ND	ND	ND	ND	ND	0.02	0.02	ND	ND	ND
16	ND	ND	ND	ND	ND	ND	0.02	0.02	0.02	0.02	0.02
17	ND	ND	ND	ND	0.02	0.02	0.02	0.02	0.02	ND	0.02
18	0.07	0.066	ND	0.042	0.007	0.02	0.02	0.02	ND	ND	0.02
19	0.03	0.2	0.09	ND	0.02	0.02	ND	ND	ND	ND	0.02
20	0.053	0.04	0.04	ND	0.02	0.02	ND	0.02	0.02	ND	ND
21	ND	0.15	0.15	0.04	0.03	0.02	ND	0.02	0.02	ND	ND
22	0.08	ND	0.06	ND	ND	ND	ND	ND	ND	ND	ND

*ND*  Not done; patients 1–11 only had data up to 18 months as part of study procedures.

Creatine Kinase in Blood-Muscle/Brain (CK-MB) was obtained and all patients had elevations noted. No consistent changes from baseline in mean CK-MB values were observed in any of the studies, and most patients with elevated post-treatment CK-MB test results had elevated baseline values.

##### Data from long-term follow up studies, registry and post-marketing reports

Approximately 2269 patients have been dosed cumulatively since OA’s first approval in May 2019 through global commercial and early access programs as of 23-May-2022. A total of 418 ICSRs containing at least one preferred term indicative of a cardiac event were identified from all sources including the long-term follow up study from START (5 reports in 5/13 patients) and no cardiac AEs were identified from the other long-term follow study. Atrial or ventricular septal defect was reported in 9 of 418 cases.

The most commonly reported cardiac adverse events were elevated cardiac troponin I, troponin T or unspecified troponin. Overall, 191 (45%) case reports included elevated cardiac troponin as a reported cardiac event. In 54% of reported cases (227/418), cardiac events were reported without associated elevations in cardiac troponin values. One hundred thirty one of 418 (31%) cases had reports of elevated cardiac troponin without any other cardiac abnormalities associated with the cardiac troponin elevation. In other words, they were isolated, asymptomatic elevations in cardiac troponin. The degree of elevations is not necessarily known due to limitation in completeness of data provided given the real-world setting. For the remainder of cases, cardiac troponin elevations with other signs/symptoms were reported in 60/418 (14%) but none of those signs/symptoms were of cardiac etiology (further discussed below). The 418 case reports are summarized in Table 10.

**Table 10:** Summary of cardiac events from post-marketing sources.

Description of cardiac events	Non-fatal case reports	Fatal case reports (any cause of death)	Total ICSRs
Troponin elevation only	129	2	131
Troponin elevation with other cardiac events	55	5	60
Cardiac events without Troponin elevation	202	25	227
Total ICSRs	386	32	418

Of the 418 ICSRs, 32 cases included an outcome of patient death. Of these 32 reports, the causes of death in 29 patients were respiratory in nature, consistent with complications of the underlying SMA. Of the 3 remaining cases, one patient (age 19 months) died of complications after acute shock (including cardiovascular collapse) resulting from prolonged bleeding events due to traumatic insertion of nasogastric tube in the setting of thrombocytopenia. Another patient (age 4 months) initially was reported to have thrombotic microangiopathy (TMA) approximately one week after OA administration. While TMA was noted to have recovered, the patient ultimately developed septic shock resulting in multi-organ system failure (including cardiac failure) and death. For the third patient, (age 15 months) reported clinical details indicated pre-existing and recurrent infections progressed to sepsis. Multiple organs including the heart were involved, with reported myocarditis, with a fatal outcome. The reported cardiac troponin, post-cardiopulmonary resuscitation was 8210 ng/mL.

The second commonly reported “cardiac event”, other than elevated cardiac troponin, identified by the given search criteria was dyspnea (reported in 95 ICSRs) which were assessed as more likely secondary to the underlying SMA. The other commonly reported events were tachycardia (in 54 case reports) or increased heart rate (in 20 case reports) which were more reported as likely secondary to compromised respiratory function, feeding difficulties or respiratory infections commonly seen in children with SMA.

While events of depressed cardiac function were reported, none of these events had a primary cardiac etiology. Such events included cardiac arrest (in 16 cases), cardio-respiratory arrest (in 5 cases), cardiac failure (in 1 cases), Cardiac failure congestive (in 1 case) and left ventricular dysfunction (in 1 case). In some cases, more than one such events were reported. In each of the reports, clinical conditions contributing to the events of depressed cardiac function included a respiratory event, multiorgan failure following delayed medical intervention of bleeding events due to thrombocytopenia (discussed for fatal cases), and fluid overload.

Myocarditis was reported in 3 cases, one of which is presented above (death event). In the second case, the patient had recurrent bacterial infection, respiratory infections, “persistent tachycardia and respiratory distress” requiring antibiotics and intubation. Baseline cardiac troponin was not available. Post-treatment cardiac troponin (not specified) was 63.5 pg/mL (reference: lower than 20) and then decreased to 3.3 pg/mL 10 days later. In the third case, the patient had a “lower respiratory tract infection” with elevated cardiac troponin I (80 ng/l, reference range not provided). Electrocardiogram and echocardiogram were reported as “normal”. The reporter indicated that “presumably this reflected a viral infiltration causing a myopericarditis or similar”.

Pericardial effusion and myopericarditis were reported in three and one cases, respectively. In the first case, baseline cardiac troponin was not reported. After OA administration, cardiac troponin (unspecified) values were available starting from week-1 with values fluctuating between 50 s – 60 s (reference <19, unit was not reported) for 2 months. Two and half months after OA administration, rhinorrhea was reported with cardiac troponin increased to 84, One week later, followed by pericardial effusion No infection was reported and COVID-19 testing was negative. Leukocyte counts were available and stable through the onset of rhinorrhea and were within normal range. No other information was available. In the second case, pericardial effusion was reported with ascites and edema. Total protein was reported as “abnormal”. Liver and renal function tests were reported as normal. In the third case, pericardial effusion was reported on an unknown date with body temperature of 38 degree Celsius. Troponin T was 0.085 ng/mL (reference range 0-0.14). In the fourth case, myopericarditis was reported on an unknown date. Troponin I was 80 ng/L (no reference available). Due to lack of clinical details, these four cases could not be further evaluated.

Based on further analysis, two-thirds of the reports of elevated cardiac troponin had concurrent reports of hepatic adverse events, most of which were abnormal liver functional tests (Table 11).

**Table 11:** Description of cardiac events with and without concurrent hepatic events.

Description of cardiac events	With hepatic event	Without hepatic event	Total ICSR
Cardiac case without troponin increase	93	134	227
Cardiac case with troponin increase	127	64	191
Troponin increase with other cardiac AE	48	12	60
Troponin increase without other cardiac AE	79	52	131
Total ICSR	220	198	418

## Discussion

Gene replacement therapy represents a novel approach to treat monogenic disorders. As with any new pharmaceutical product, all sources of safety data are evaluated to elucidate and further characterize potential and documented safety risks.

Although SMA is traditionally known as a motor neuron disease predominantly affecting the ventral horn of the spinal cord, SMA patients often present with multiple structural heart malformations as well as ECG abnormalities [5, 9, 10]. Numerous clinical studies have suggested an increased frequency of cardiac problems in SMA patients, such as arrhythmias, cardiomyopathy, valvular aortic stenosis, hypoplastic aortic arch, severe coarctation of the aorta, partial atrioventricular canal, tricuspid atresia, univentricular heart, and hypoplastic left heart [4, 5, 11, 12]. Coletta and colleagues (1989) also observed ECG abnormalities in the absence of structural cardiac defects in 12 of 13 SMA patients and concluded that the abnormalities were due to fasciculations of denervated muscle [10]. Similarly, isoelectric line tremors without changes in heart rhythm or function were reported by Huang and colleagues, signifying likely neuronal re-innervation activity during the course of alpha motor neurons resulting in muscle fasciculation [13]. Evidence on ECG of right ventricular overload, possibly caused by pulmonary hypertension due to respiration abnormalities in patients with SMA, has also been described. Therefore, it is important for clinicians to evaluate the presence of underlying or evolving cardiac disease in patients with SMA.

Nonclinical cardiovascular toxicity findings that could potentially be relevant to the clinical use of OA were observed in 2 separate mouse GLP toxicology studies. In these mouse studies, IV administration of OA resulted in heart findings that were consistent across studies and dose-related. Findings in the ventricles of the heart of these mice were composed mainly of inflammation, edema, and fibrosis; and showed evidence of maturation from an early event predominated by inflammation, with gradual maturation of the reaction to yield predominantly fibrosis from weeks 3 to 12 postdose. Similar myocardial findings were occasionally observed in the atrial myocardium, but the dominant atrial finding was atrial thrombosis at doses ≥2.4 × 10^14^ vg/kg, which are significantly higher than the therapeutic human dose (1.1 × 10^14^ vg/kg). While perinatal mice are considered representative for the very young neonatal patient population receiving OA, developmentally, rodents are considered more mature at birth than humans, undergoing very rapid postnatal maturation. Mice at 3 to 7 weeks of age are considered developmental equivalent to a child or adolescent and have fully mature hematologic and blood coagulation functions. The occurrence of thrombosis at this age in mice would not be attributable to functionally immature coagulation and hemostasis.

Administration of OA to juvenile NHPs was generally well tolerated with no elevated plasma cTnI and no heart-related pathological findings at up to 6 months following IV administration at 1.1 × 10^14^ vg/kg. To date, no evidence of intracardiac thrombi has been observed in humans. Based on the reported adverse events, as well as objective data reported, there is no evidence of overt and direct cardiac toxicity due to OA treatment in patients.

Electrocardiographic abnormalities (and in some cases in the absence of structural cardiac defects) have been described for SMA patients, and can be a background finding for SMA patients. In NHPs administered OA, no changes in ECG parameters including PR interval, QRS duration, QTc, and heart rate were observed at up to 6 months following IV administration at 1.1 × 10^14^ vg/kg. In SMA patients administered OA, ECG findings did not show evidence of pathology, specifically no arrhythmias or abnormalities in PR interval, QRS duration, QTc. Some patients had reported events of tachycardia or bradycardia secondary to alternate etiologies such as fever and respiratory dysfunction. Further, echocardiography evaluation of LVEF and LVSF indicated normal cardiac systolic function in all patients studied in clinical trials, without presence of thrombi. Due to the lack of clinical signals from both NHPs and humans routine ECG or echocardiograms monitoring after OA administration is not warranted.

Creatine Kinase in Blood-Muscle/Brain is one of the 3 creatine-kinase isoenzymes expressed in the heart (approximately 22% of the total creatine kinase content) and skeletal muscle (approximately 5 to 7%) [14]. CK-MB elevations were monitored in the clinical trials and were not considered meaningful as all patients in the clinical trials had elevations and CK-MB are known to be elevated in children SMA. Further, no consistent changes from baseline in mean CK-MB values were observed in any of the studies, and most patients with elevated post-treatment CK-MB test results had elevated baseline values. Hence, CK-MB values have no applicability in monitoring children treated with OAV101.

Cardiac troponin comprises a group of cardiac specific proteins which are released from cardiac cardiac myocytes into the circulation after myocardial injury. Cardiac troponins are readily measurable, however, there are different isoforms of cardiac troponin (cardiac troponin I and cardiac troponin T), which vary in their specificity and sensitivity. This variability in isoform measurements further complicates interpretation and reliability as specificity and sensitivity of assays can vary and is a limitation of the data obtained in humans after OA dosing [15]. Cardiac troponins, particularly cardiac troponin T, can also be elevated in critically ill patients with various disease entities including cardiac and non-cardiac etiologies such as myocarditis, sepsis, cardiotoxic medications and chronic kidney disease [16–18]. Further, cardiac troponin values in children can be challenging to interpret in isolation as normative values have not been established and reference ranges in children may vary [19]. Yoldas et al. (2019) described the most common causes of elevated cardiac troponin I (>0.06 ng/mL) in over 750 children who did not have a history of congenital heart disease, heart surgery, sepsis or neonatal asphyxia who underwent cardiac troponin testing because of check pain, syncope, CHF, palpitation and suspected arrhythmia [20]. The authors reported that primary cardiac etiologies comprised approximately 47% of causes for cardiac troponin elevations; however, these patients were symptomatic. Other associated pathologies included drug intoxication (21%), carbon monoxide intoxication (18%), bronchopneumonia-asthma (175), shock (11%), sepsis (10%), convulsive-status epilepticus (8%), and asphyxia (8%). Further, normative values in chronic medical conditions, especially with chronic pulmonary involvement and potential cardiac involvement have not been determined. In approximately 2269 OA-treated children with SMA, based on the reported adverse events to date, cardiac troponin elevations were seen either in association with these non-cardiac etiologies, namely bronchopneumonia as well as sepsis in a few cases, or in most cases without any recognizable clinical relevance. Further, none of the fatal cases represented primary cardiac etiology. These data suggest that patient factors may be contributory with respect to the elevations seen in humans. Furthermore, the cardiac troponin elevations seen in the reported AEs after OA-treatment in children with SMA were mainly asymptomatic in nature. From the available clinical reports, none of the cardiac troponin elevations represented primary cardiac pathology, which is consistent with the Yoldas report, and is likely associated with other factors, including possible intrinsic effects to the underlying SMA or concomitant disease process. The cardiac troponin data presented has limitations; however, even if real, the changes were minor, with no significant clinical manifestations, were all reversible without treatment and limited by comorbidities and potential effects of liver transaminitis in cTn clearance. This raises the possibility of cardiac troponin elevations occurring from cardiac uptake of OA, thereby providing another possible etiology for transient elevations in children dosed with OA.

Interestingly, in the human data, after treatment with OA, most if not all abnormal cardiac troponin levels have been accompanied by transaminitis with abnormal alanine aminotransferase (ALT) / aspartate aminotransferase (AST) in the setting of liver injury/hepatitis. Data from some studies suggested that elevations of cardiac troponin levels is common in patients with both acute and subacute liver failure and hence, might signify a secondary myocardial injury in a state of metabolic stress in the setting of multi-organ involvement [21, 22]. Furthermore, limited literature indicates cardiac troponin clearance may be affected by liver function, specifically, due to abnormal scavenger receptor clearance resulting in stable elevations of cell damage biomarkers, including cardiac troponin [23]. As the liver is also a target organ of toxicity for OA, the increases in cardiac troponin in the setting of transaminitis in some SMA patients, may be associated with a decrease in cardiac troponin clearance. The possible decrease in cardiac troponin clearance in the setting of acute transaminitis could support a postulation that increased uptake of OA in the liver occurs transiently, thereby resulting in elevations of hepatic aminotransferases from hepatic injury with a secondary increase in cardiac troponin. This phenomenon could be potentiated in children with SMA given the cardiac and liver involvement associated with the underlying disease, which could further explain why cardiac troponin elevations were not seen in healthy NHP studies [24]. Finally, adenovirus can cause myocarditis and it cannot be ruled out that the vector may induce mild transient cardiac inflammation in a small subgroup of patients, but this is not supported by the available human data in patients treated with OA [25].

In summary, based on cardiac findings documented in mice, cardiac troponin monitoring has been recommended in product labeling after OA dosing. The results in NHP toxicology studies, together with the currently available clinical data (clinical studies and post-marketing) puts into question the translatability of the rodent findings into humans, and does not support the hypothesis of a direct and significant OA-induced cardiotoxicity in humans. SMA can be associated with cardiac events due to underlying disease and secondary complications. Healthcare professionals should use medical judgment when evaluating the etiology and assessment of cardiac events post OA dosing so as to consider all possibilities and manage the patient accordingly.

## Data Availability

Due to its proprietary, supporting data cannot be made openly available. Further information about the data and conditions for access are available at the Novartis Pharma.
